# Cumulative Midlife Neighborhood Disadvantage and Late-Life Cognitive Change: The Mediating Role of Hemoglobin A1C

**DOI:** 10.1093/geroni/igaf122.745

**Published:** 2025-12-31

**Authors:** Greta Jianjia Cheng, Jeanine Buchanich, Tiffany Gary-Webb, Christina Mair, C Shaaban, Andrea Rosso

**Affiliations:** University of Pittsburgh, Pittsburgh, Pennsylvania, United States; University of Pittsburgh, Pittsburgh, Pennsylvania, United States; University of Pittsburgh, Pittsburgh, Pennsylvania, United States; University of Pittsburgh, Pittsburgh, Pennsylvania, United States; University of Pittsburgh, Pittsburgh, Pennsylvania, United States; University of Pittsburgh, Pittsburgh, Pennsylvania, United States

## Abstract

Neighborhood factors are recognized determinants of late-life cognitive function; mechanisms remain unclear but may include metabolic factors. Using data from the Health and Retirement Study, we examined whether hemoglobin A1C, a 3-month measure of blood glucose level, partially explains the association between cumulative midlife neighborhood disadvantage and late-life cognitive change in 4,707 older adults aged 50+. Cumulative midlife neighborhood disadvantage (continuous score) captured average exposure to tract-level disadvantage, including poverty, unemployment, % female-headed households, and % households with public assistance. A1C level was categorized as < 5.7%, 5.7%-6.5%, and > =6.5%. Cognitive function was repeatedly assessed using the Telephone Interview for Cognitive Status. Cognitive change groups (maintainers, minor decliners, major decliners) were identified based on participant-specific random slopes from a linear mixed model. Path analyses estimated coefficients and product method calculated the mediation effect. Higher neighborhood disadvantage was associated with elevated A1C (5.7%-6.5%, RRR—relative risk ratio: 1.2, 95% CI: 1.1, 1.4; > =6.5%, RRR: 1.4, 95% CI: 1.2, 1.6), and was associated with minor (RRR: 1.34, 95% CI: 1.16, 1.55) or major cognitive decline (RRR: 1.71, 95% CI: 1.43, 2.03). After controlling for A1c, neighborhood disadvantage remained associated with cognitive change, suggesting a partial mediating effect from blood glucose. Controlling the A1C level from > =6.5% to < 5.7% would potentially reduce the adverse effect of neighborhood disadvantage by 12% for minor and 16% for major cognitive decline. Midlife is a critical period of neighborhood exposure for cognitive health; targeting blood glucose could partially mitigate the adverse effect of neighborhood disadvantage on late-life cognitive decline.

